# pH-Responsive Delivery of H2 through Ammonia Borane-Loaded Hollow Polydopamine for Intervertebral Disc Degeneration Therapy

**DOI:** 10.1155/2023/7773609

**Published:** 2023-02-02

**Authors:** Weiheng Wang, Bing Xiao, Yuanyuan Qiu, Yi Liu, Guoke Tang, Guoying Deng, Yanhai Xi, Guohua Xu, Yeying Wang

**Affiliations:** ^1^Department of Orthopaedics, Second Affiliated Hospital of Naval Medical University, No. 415 Fengyang Road, Shanghai, China; ^2^School Hospital of Shanghai University of Sport, No. 399, Changhai Road, Shanghai, China; ^3^Department of Orthopedics, Shanghai General Hospital, Shanghai Jiaotong University, No. 100 Haining Road, Shanghai, China; ^4^Trauma Center, Shanghai General Hospital, Shanghai Jiaotong University School of Medicine, No. 650 Xin Songjiang Road, Shanghai, China; ^5^Medical Frontier Innovation Research Center, The First Hospital of Lanzhou University, No. 11 Donggang West Road, Lanzhou, China

## Abstract

An imbalance in oxidative and inflammatory regulation is the main contributor to intervertebral disc degeneration (IDD). Hydrogen (H2) therapy is a promising antioxidation and anti-inflammatory approach. However, the key to the treatment is how to maintain the long-term effective H2 concentration in the intervertebral disc (IVD). Therefore, we developed a pH-responsive delivery of H2 through ammonia borane-loaded hollow polydopamine (AB@HPDA) for IDD therapy, which has sufficient capacity to control long-term H2 release in an acid-dependent manner in degenerative IVD. The characterization, toxicity, and pH-responsive H2 release of AB@HPDA was detected in vitro. The metabolization of AB@HPDA in the degenerated IVD was tested by in vivo imaging. The therapeutic effect of AB@HPDA on IDD was tested in vivo by X-ray, MRI, water content of the disc, and histological changes. Nuclear extracellular matrix (ECM) components, oxidative stress, and inflammation were also tested to explore potential therapeutic mechanisms. AB@HPDA has good biocompatibility at concentrations less than 500 *μ*g/mL. The H2 release of AB@HPDA was pH responsive. Therefore, AB@HPDAs can provide efficient hydrogen therapy with controlled H2 release in response to the acidic degenerated IVD microenvironment. The metabolization of AB@HPDA in IVD was slow and lasted up to 11 days. HPDA and AB@HPDA significantly inhibited IDD, as tested by X-ray, MRI, disc water content, and histology (*P* < 0.05). pH-responsive H2 delivery through AB@HPDAs has the potential to efficiently treat IDD by inhibiting ECM degradation and rebalancing oxidative stress and inflammation in degenerative IVDs.

## 1. Introduction

With changes in modern lifestyles and the aging of the population, the incidence of neck and low back pain is increasing year by year. This imposes a heavy economic burden on society [1, 2]. Intervertebral disc degeneration (IDD) is considered the most common cause of neck and low back pain [3]. Neck and low back pain currently affects approximately 70% of the global population and is a leading cause of disability, loss of productivity, and decreased quality of life [4]. Although factors such as aging, weight-bearing, trauma, smoking, hyperglycemia, and genetics are associated with IDD, the pathogenesis of IDD remains unclear [5]. Current treatments for IDD, including drug therapy and surgery, are aimed at relieving clinical symptoms. Contemporary treatments cannot reverse or delay the process of IDD, and some even accelerate IDD. For example, discography [6] and discoblock [7, 8] can significantly increase IDD. Intervertebral fusion surgery can increase IDD in adjacent segments, leading to the occurrence of adjacent segment disease (ASD) [9]. Therefore, an effective treatment to reverse the progression of IDD is urgently needed.

At present, there is no effective treatment to stop the progression of IDD, which is a major bottleneck in the treatment of IDD. One of the factors influencing therapeutic efficacy is that it is difficult to maintain the long-term effective drug concentration in the intervertebral disc (IVD) with conventional drug delivery methods. The IVD is an enclosed space and is the largest nonvascular tissue in the body. Studies have found that IVD nutrient metabolism mainly occurs through the passive diffusion of endplate cartilage (EP), which is significantly lower than that of other tissues. With increasing age and damage to the EP, the passive diffusion capacity decreases significantly, leading to IDD [10]. Therefore, it is difficult to form an effective drug concentration in IVD by conventional routes of administration (oral, intravenous, intraperitoneal, etc.), which greatly reduces the therapeutic effect of IDD. For the above reasons, the treatment cycle for intervertebral disc infections is longer and less effective [11]. While increasing the drug concentration may enhance its efficacy, high doses increase the incidence of side effects. Disc puncture for local drug delivery is often used in clinical and research applications [12–14]. Repeated disc puncture is often required to address the short half-life of the drug. Disc puncture can significantly exacerbate IDD, especially repeated puncture [7, 15, 16]. The special microenvironment of low oxygen, high pressure, and high acidity in the IVD is another bottleneck for IDD treatment [10]. Normal tissues have an extracellular pH of 7.4[17]. The pH in healthy IVD is approximately 7.1, while the pH in mildly, moderately, and severely degenerated IVD are reported as 6.8, 6.5, and 6.2, respectively [18]. IDD exacerbates the acidic environment in the IVD. The acidic environment of IVD reduces the activity and efficacy of drugs to treat IDD [19]. In our previous study of stem cell transplantation in IDD, it was found that the special microenvironment in the IVD led to a low survival rate of transplanted cells and poor treatment effect [12, 13].

Oxidative stress injury plays an important role in IDD, so reducing reactive oxygen species levels may be a therapeutic target for IDD. Studies have found that reactive oxygen species (ROS) levels increase dramatically in IDD, and redox homeostasis and inflammatory imbalance play important roles in IDD [20]. Studies have shown that elevated levels of ROS (including nitric oxide, peroxynitrite, hydrogen peroxide, hydroxyl radicals, and lipid hydrogen peroxide) can promote nucleus pulposus cell (NPC) apoptosis, reduce extracellular matrix (ECM) synthesis, and ultimately lead to IDD [21]. Therefore, decreasing the expression of ROS may alleviate IDD. Hydrogen (H2) therapy, as an emerging and promising strategy for alleviating oxidative stress, has attracted extensive attention due to its antioxidant and anti-inflammatory effects [22]. H2 possesses excellent biopermeability and biosafety and has been shown to attenuate intracellular ROS-induced cytotoxicity and inflammatory responses [22]. H2 has shown good therapeutic effects in the treatment of various diseases [23–25]. Conventional H2 administration methods include direct inhalation and the use of hydrogen-rich water. However, due to the rapid removal of H2 donors from the lesion and the rapid volatilization of H2 gas, the H2 concentration in the lesion area will decrease rapidly, failing to achieve the goal of long-term treatment. In addition, due to the closed microenvironment in the IVD, it is difficult to maintain the effective H2 concentration for a long time by oral inhalation or intravenous infusion, which severely limits the application of H2 in IDD. There are no reports of H2 in the treatment of IDD thus far.

To achieve efficient delivery and endogenous stimulus-controlled long-term release in the IVD, we recently developed a pH-responsive ammonia borane (AB) as a precursor for H2 production. AB has a good H2 storage capacity (10 wt.%) and can be decomposed into H2 in an acid-dependent manner [26]. Both normal and degenerated discs have an acidic environment due to the accumulation of byproducts of glycolysis, such as lactic acid. Acidity levels are associated with the grade of IDD [18, 27]. AB can efficiently release H2 in an acid-responsive manner, and as IDD levels increased, so did the ability of AB to release H2. To increase the H2-controlled release effect of AB, we adopted hollow polydopamine (HPDA) as the carrier of AB and designed a pH-responsive delivery of H2 through ammonia borane-loaded hollow polydopamine (AB@HPDA). HPDA has the characteristics of a large surface area, good biocompatibility, and strong permeability. We hypothesized that AB@HPDA might treat IDD by inhibiting excess ROS production and modulating inflammation.

To the best of our knowledge, this is the first proposal of pH-responsive H2-releasing nanomaterials designed for IDD treatment based on the specific acidic environment of IVD. In this study, we successfully prepared AB@HPDA nanoparticles and injected them into degenerated IVDs in rats. We performed a detailed in vitro evaluation of the functional properties of AB@HPDA, including H2 release kinetics and biocompatibility. Then, we investigated the metabolism of AB@HPDAs in degenerated IVDs and the therapeutic effect and possible mechanism of IDD in vivo. In vivo experimental studies showed that AB@HPDAs could significantly inhibit IDD by reducing oxidative stress and inflammation and promoting extracellular matrix (ECM) secretion. pH-responsive delivery of H2 through AB@HPDA nanoparticles is a promising strategy for the treatment of IDD.

## 2. Materials and Methods

### 2.1. Synthesis and Characterization of AB@HPDA Nanoparticles

First, monodisperse silicon dioxide nanoparticles (SiO2) were synthesized according to the typical Stöber method [28]. Briefly, 74 mL ethanol, 3.14 mL NH3·H2O, and deionized water were stirred at 30°C for 10 min. Next, 3.5 mL tetraethyl orthosilicate (TEOS) was added dropwise to the reaction flask, and stirring was maintained for 1 h. SiO2 was obtained by centrifugation and redispersed in 30 mL of deionized water for later use. Subsequently, the above SiO2 (5 mL) was dispersed in 40 mL Tris buffer (pH = 8.5), and then 2.5 mL dopamine hydrochloride solution (DA, 75 mg/mL) was added rapidly and stirred at room temperature for 9 h to obtain SiO2@PDA nanoparticles. Next, the obtained SiO2@PDA was placed in a mixture of 30 mL ethanol and 30 mL deionized water, and 6 mL HF solution was added while stirring and allowed to stand overnight to obtain hollow PDA (HPDA) nanoparticles. Finally, AB molecules were dissolved in HPDA aqueous solution and stirred for 24 h at room temperature to obtain AB@HPDA nanoparticles. The SiO2@PDA and HPDA nanoparticles were characterized by transmission electron microscopy (TEM) (JEM-2100F, Tokyo) and scanning electron microscopy (SEM) (Philips XL30-FEG). The high-angle annular dark field (HAADF) scanning TEM (STEM) imaging was used to monitor the composition of AB@HMPDA nanoparticles. A Super-X detector was applied to the energy dispersive X-ray mapping in a STEM mode (STEM-EDX).

### 2.2. Animal Model of IDD

Sprague–Dawley (SD) rats were used to establish the IDD model in this study. All animals were purchased from the Experiment Animal Center of Naval Medical University (Shanghai, China). The animals (two per cage) were housed in pathogen-free conditions. All rats were placed at 20°–24°C, 40%-70% relative humidity, 12 h/12 h day-night cycle and received food and water ad libitum. They were allowed at least 1 week to acclimate to the environment before all experimentation. Animal experimental protocols were approved by the Animal Ethics Committee of Naval Medical University, and all experiments were carried out in accordance with the Guide for the Care and Use of Laboratory Animals.

A total of 80 Sprague–Dawley rats (280-300 g) were used. In vivo experiments were performed on 3-month-old male rats. Rats in this age group are generally considered skeletally mature [29]. Rats were randomly assigned to four experimental groups, including the sham group, IDD group, HPDA group, and AB@HPDA group (*n* = 20). Our previous study confirmed that IDD was closely related to the diameter of the needle. Therefore, we chose a 21G needle to establish the rat IDD model and a 30G 4needle to inject different solutions [6]. The IDD models were established as described by Rousseau et al. [30]. Co7–Co8 coccygeal discs of rats were punctured by a 21G needle after anesthetization with isoflurane. A 21-gauge needle was inserted parallel to the endplate, 3.0 mm into the disc, where it was rotated 360° and held for 30 s. Rats in all groups except the sham group received acupuncture procedures. The sham group received the same preoperative preparation but without puncture. Two weeks later, 2 *μ*L saline was injected into Co 7/8 in IDD, 2 *μ*L HPDA was injected into Co 7/8 in the HPDA group, and 2 *μ*L AB@HPDA (500 *μ*g/mL) was injected into Co 7/8 in the AB@HPDA group. The sham group remained intact. The liquid sample was injected slowly and steadily over 3 minutes using a microliter syringe (Hamilton, Switzerland). The injection needle was left in the IVD for at least 2 minutes to prevent leakage after injection. We defined the injection time (2 weeks after modeling) as the starting time (0 weeks). Then, IDD was evaluated by imaging and histological analysis.

### 2.3. Assessment of the Release Kinetics of H2 In Vitro

The pH responsiveness of AB@HPDA at 37°C was investigated by measuring the kinetics of H2 evolution from AB@HPDA nanoparticles under different conditions. To examine the effect of pH, phosphate buffered solutions (PBS) were prepared at five different pH values (pH = 6.2, 6.5, 6.8, 7.1, and 7.4). The 10 mg AB@HPDA nanomedicines were uniformly dispersed in 100 mL of n2-saturated PBS with different pH values (6.2, 6.5, 6.8, 7.1, and 7.4), and the reaction vessel was immediately sealed. The H2 content in solution was determined by gas chromatography (GC2060).

### 2.4. Evaluation of AB@HPDA Biocompatibility

Extraction and cultivation of NPCs were performed as described in our previous study [12]. NPCs were cultured in complete culture medium including F12/DMEM, 10% fetal calf serum (FBS, Gibco, Thermo Fisher Scientific, USA), and 1% penicillin/streptomycin (Gibco, Thermo Fisher Scientific, USA). NPCs were seeded onto six-well plates at a density of 1 × 10^5^ cells/well and cultured in a cell culture incubator at 5% CO_2_ at 37°C. After incubation for 24 h, the media was replaced with fresh media containing different concentrations of HPDA or AB@HPDA (0 *μ*g/mL, 50 *μ*g/mL, 200 *μ*g/mL, 500 *μ*g/mL, and 1000 *μ*g/mL). According to the instructions of the calcein AM/propidium iodide (PI) Kit (Yeasen Biotechnology, Shanghai, China), dead and living cells were stained. Here, living cells were stained with green fluorescent calcein acetoxymethyl ester (calcein AM), and dead cells were stained with red fluorescent propidium iodide (PI).

### 2.5. Evaluation of AB@HPDA Accumulation and H2 Generation In Vivo

In vivo AB@HPDA nanoparticle accumulation was measured by ex vivo imaging. Based on the live/dead staining results, 500 *μ*g/mL AB@HPDA showed minimal cellular cytotoxicity. To trace the nanoparticles in the intervertebral disc area, AB@HPDA (500 *μ*g/mL) nanoparticles were labeled with Cy5.5. The labeled nanoparticles (500 *μ*g/mL, 2 *μ*L) were injected into the nucleus pulposus region of the intervertebral disc using a 30G needle. Images were taken at specified time points (0 h, 1 h, 12 h, 1 d, 3 d, 5 d, 7 d, 9 d, 11 d, and 13 d) using an IV Scope 8000 imaging system (Shanghai CLINX Science, China). The generation of H2 in vivo was measured by a gas chromatograph device (Gas Chromatography-9860; Qiyang Co.) using the H2 concentration in venous blood.

### 2.6. X-Ray and MRI

At 0, 4, 8, and 12 weeks after injection, the rats were anesthetized with pentobarbital (3%) to relax the tail muscles of the rats (*n* = 10). After anesthesia, the rats were placed in the prone position for X-ray and MRI scans. The change in DHI detected by X-ray was expressed as DHI % according to the method of Han et al. [31]. The degree of IDD was evaluated on magnetic resonance imaging (MRI, 3.0 T; United Imaging, Shanghai, China) based on the signal intensity in sagittal T2-weighted images. All images were analyzed by two independent observers who were blinded to the experimental conditions.

### 2.7. Disc Water Content

At 12 weeks after injection, all rats were sacrificed by pentobarbital overdose (*n* = 10). Co7/8 was separated from adjacent vertebrae with a scalpel and frozen in liquid nitrogen for 10 minutes. The wet weight (WW) of the specimens was measured using an electronic balance (HA125SM, Precisa, Dietikon, Switzerland) with an accuracy of 0.01 mg. Then, the specimens were dried in an oven at 65°C for 24 hours. The dry weights (DW) were recorded in the same manner. The disc water content was calculated as [(WW − DW)/WW] × 100%.

### 2.8. Histological Analysis

At 12 weeks after injection, Co7/8 discs were harvested for histological analysis (*n* = 5). Collected samples were fixed with 4% paraformaldehyde and then decalcified and embedded in paraffin in sections. All specimens were sectioned at 5 *μ*m sagittally. Hematoxylin and eosin (H&E) staining, safranin O-fast green (S-O) staining, and Masson staining were performed. The histologic scores were evaluated by the method adapted from Han et al. [31]. According to this method, the cellular structure and morphology of AF and NP and the boundary between the two structures were evaluated. Histologic scores range from 5 to 15, with higher scores indicating more severe disc degeneration. Normal discs are awarded 1 point for each category, while discs representing severe degeneration are awarded 3 points for each category.

### 2.9. Western Blotting

The expression levels of collagen II, aggrecan, matrix metalloproteinase 13 (MMP-13), and tissue inhibitor of metalloproteinase 1 (TIMP-1) in degenerative nucleus pulposus (NP) tissues were measured using western blotting. At 12 weeks after injection, the NP tissue of Co 7/8 was harvested and frozen in liquid nitrogen (*n* = 5). The NP tissue was fully shredded after weighing, and the tissue was homogenized by a homogenizer. Then, they were lysed by adding ice-cold RIPA lysis buffer. The collected tissue homogenate was centrifuged at 14000 × g at 4°C for 10 min, and the supernatant was collected. Protein concentrations were measured using a BCA protein assay reagent kit (Pierce, Rockford, Milwaukee, WI, USA). Equal amounts of protein were then separated through 12% sodium dodecyl sulfate–polyacrylamide gel electrophoresis (SDS–PAGE), after which they were transferred onto nitrocellulose membranes (Invitrogen, Carlsbad, CA, USA). The membranes were then incubated overnight at 4°C with the following antibodies: collagen II (1 : 2000; AF0135, Affinity), aggrecan (1 : 2000; DF7561, Affinity), MMP-13 (1 : 3000; 18165-1-AP, ProteinTech), and TIMP-1 (1 : 1000; sc-21734, Santa Cruz). Next, the HRP-conjugated antibody (1 : 10000; ab6747, Abcam) was used as the secondary antibody. Protein bands were visualized using an enhanced chemiluminescence system (Thermo Fisher Scientific). Quantification of western blotting results was performed using the ImageJ software, version 1.8.0.

### 2.10. Detection of Superoxide Dismutase (SOD) and Malondialdehyde (MDA)

Twelve weeks after injection, the rats were anesthetized, and the NP tissues were harvested and frozen in liquid nitrogen (*n* = 5). The levels of SOD and MDA were determined using SOD and MDA assay kits (Jiancheng Bioengineering Institute, Nanjing, China) according to the manufacturer's instructions. Th NP tissues were lysed, and the supernatant was collected. Absorbance was measured at a wavelength of 532 nm (MDA) or 550 nm (SOD) by using a microplate reader.

### 2.11. Detection of Inflammatory Cytokines by Enzyme-Linked Immunosorbent Assay (ELISA)

Twelve weeks after injection, the NP tissues were harvested (*n* = 5). Subsequently, the NP tissues were accurately weighed using an electronic weighing machine. NP tissues were dissolved, and the homogenate was collected by centrifugation at 2000 × g for 15 min. The supernatant was collected, and the contents of IL-1*β*, IL-6, and TNF-*α* in the supernatant were detected strictly according to the instructions of the ELISA test kit (R&D Systems, Minneapolis, MN, USA) by using the kit instructions (ELx800, BioTek) at 450 nm absorbance value.

### 2.12. Statistical Analysis

Statistical analysis was performed using SPSS Standard version 21.0 (IBM, USA) and GraphPad Prism 8.0 (GraphPad, USA). All experimental data are expressed as the mean ± standard error. Statistical analysis was done comparing multiple groups with one-way analysis of variance followed by Tukey's post hoc test. All differences were considered statistically significant at *P* < 0.05.

## 3. Results

### 3.1. Characterization of the AB@HPDA Nanoparticles

**Figure 1:**
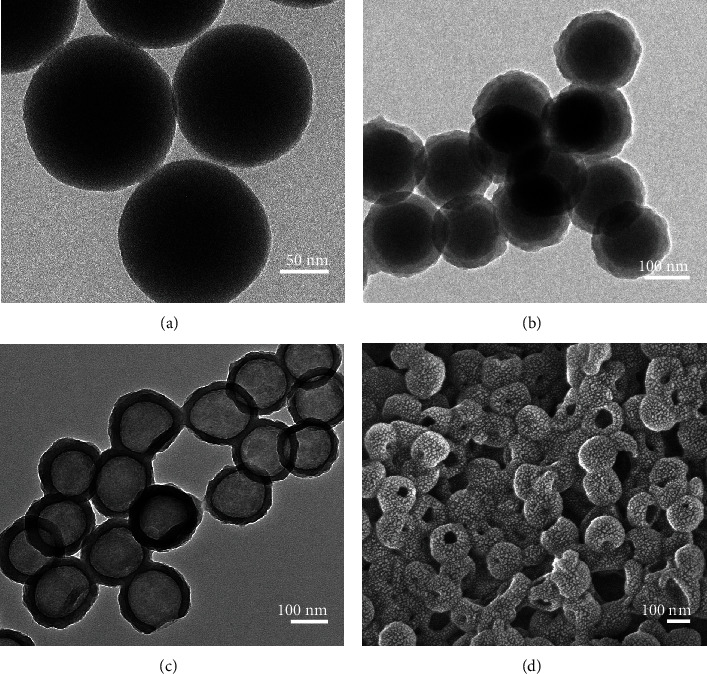
Characterization of AB@HPDA nanoparticles. TEM analysis of (a) SiO2, (b) SiO2@PDA, and (c) HPDA nanoparticles. (d) SEM images of HPDA nanoparticles.

AB was synthesized and characterized according to a previous report [32]. TEM and SEM images were used to analyze the morphology of the nanoparticles.  Figure 1 shows dense HPDA nanoparticles with a diameter of 182 nm, and the synthesized HPDA had a fairly uniform size and good monodispersity. Moreover, HPDA has a highly hollow structure, which is favorable for high drug loading and sustained drug release. To further confirm the constituents, the AB@HPDA nanoparticles were measured by energy dispersive X-ray (EDX) spectroscopic analysis matched scanning transmission electron microscopy (STEM). As shown in Figure S1, the carbon (C), nitrogen (N), and oxygen (O) elements were interwoven in whole AB@HPDA nanoparticles. In addition, the boron (B) element was interspersed in the nanoparticles, which indicated the successful synthesis of AB@HPDA nanoparticles.

### 3.2. pH-Responsive H2 Release of the AB@HPDA Nanoparticles

**Figure 2:**
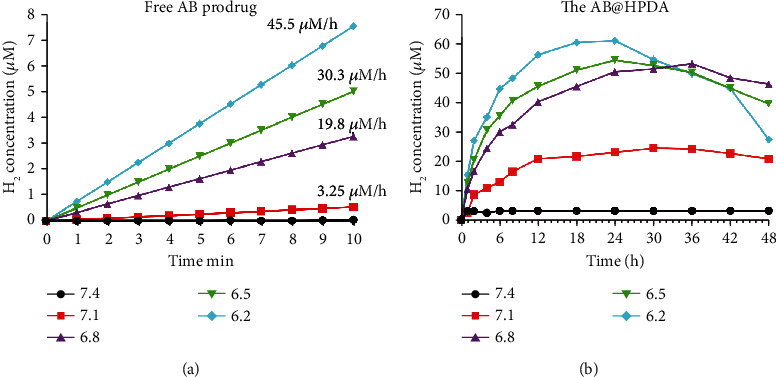
The cumulative release kinetics of H2 from the (a) porous AB and (b) AB@HPDA nanospheres in PBS (0.01 M, 37°C) at pH 7.4, pH 7.1, pH 6.8, pH 6.5, and pH 6.2 for 24 h.

The AB release mechanism for H2 is presented as NH_3_ · BH_3_ + H^+^ + 3H_2_O = NH_4_^+^ + B(OH)_3_ + 3H_2_. As shown in the reaction equation, AB can exist stably under neutral and alkaline conditions while releasing H2 in acidic pH through an intramolecular cyclization reaction. Five different pH values of PBS (pH = 7.4, 7.1, 6.8, 6.5, 6.2) were used to simulate intradiscal pH values with different degrees of degeneration, and the acid-responsive decomposition behavior of AB to H2 release was tested. As shown in Figure 2(a), free AB is rapidly decomposed to H2 in acidic PBS, and the higher the acidity of PBS is, the faster the H2 release. At pH = 7.4 PBS (simulating a normal tissue environment), free AB did not undergo significant decomposition. In the slightly acidic environment of pH = 7.1 (simulating the normal intradiscal microenvironment), the H2 release rate was only 3.25 M/h. In the acidic environment of pH = 6.8, 6.5, and 6.2 (simulating mild, moderate, and severe degeneration of the intradiscal microenvironment, respectively), the H2 release rates were as high as 19.8 *μ*M/h, 30.3 *μ*M/h, and 45.5 *μ*M/h, respectively. These results reflect the higher acid responsiveness of AB. However, free AB molecules are easily excreted from IVDs, resulting in a low therapeutic effect. To overcome this disadvantage, free AB was encapsulated into HPDA nanoparticles to prolong its retention time in the IVD. AB@HPDA nanomaterials have acid-responsive release of H2, which is similar to AB nanomaterials, as shown in Figure 2(b). This enables AB@HPDA nanomaterials to realize H2 release in response to the acidic microenvironment in the degenerative disc for efficient hydrogen therapy. In addition, the decomposition rate of AB in HPDA is greatly reduced compared with that of free AB (Figure 2). As shown in Figure 2(b), the H2 in AB@HPDA was rapidly released in the first 30 minutes, followed by a slow sustained release, and the H2 content peaked at approximately 24 hours and then decreased slowly.

### 3.3. Biocompatibility of the AB@HPDA Nanoparticles In Vitro

**Figure 3:**
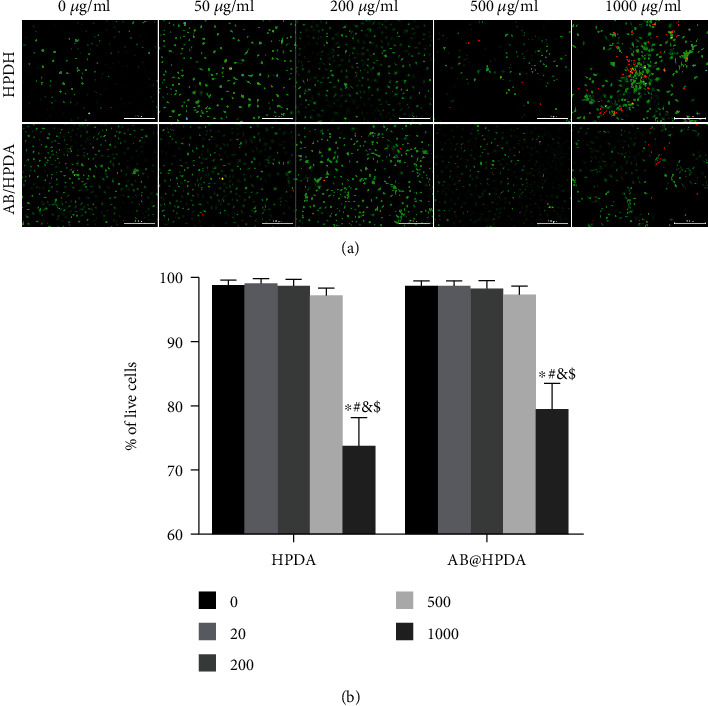
(a) Calcein AM/PI staining of NPCs under administration of HPDA or AB@HPDA at different concentrations for 1 h. (b) NPC viability was exposed to different concentrations of HPDA/AB@HPDA nanoparticles for 1 h. Data are presented here as the mean ± SD. ^∗^*P* < 0.05 vs. 0, ^#^*P* < 0.05 vs. 10, ^&^*P* < 0.05 vs. 100, ^$^*P* < 0.05 vs. 500 (*n* = 5). Scale bar = 200 *μ*m.

The toxicity of HPDA and AB@HPDA nanoparticles was assessed by calcein AM/PI staining. NPCs were cultured in HPDA and AB@HPDA nanoparticles at concentrations of 0 *μ*g/mL, 50 *μ*g/mL, 200 *μ*g/mL, 500 *μ*g/mL, and 1000 *μ*g/mL for 1 h. Live cells are stained green, and dead cells are stained red. The proportions of viable cells in the 0 *μ*g/mL, 50 *μ*g/mL, 200 *μ*g/mL, and 500 *μ*g/mL groups were >97%, and there was no significant difference between the groups (*P* > 0.05Figure 3). A large number of red-stained cells were observed in the 1000 *μ*g/mL group, and the proportions of viable cells in the HPDA and AB@HPDA groups were 73.4% and 79.2%, respectively, which were significantly lower than those in the other groups (*P* < 0.05%). Taking into account the effectiveness and biocompatibility of AB@HPDA, the appropriate concentration of AB@HPDA for in vivo treatment was determined to be 500 *μ*g/mL.

### 3.4. The Metabolization of AB@HPDA Nanoparticles in the Degenerated IVD

**Figure 4:**
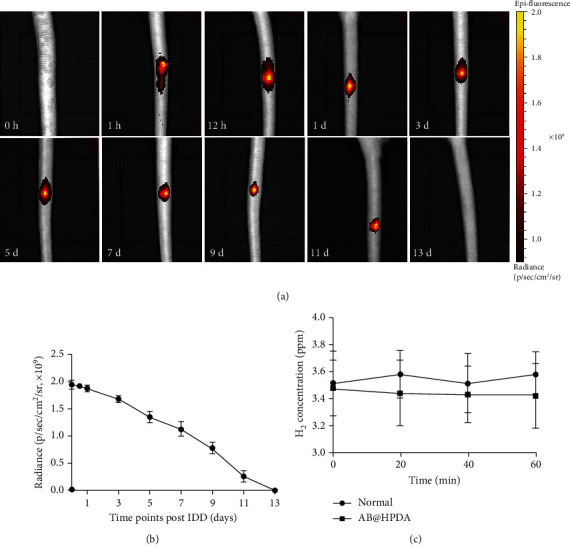
(a, b) In vivo fluorescence imaging and quantitative analysis of AB@HPDA nanoparticles labeled with Cy5.5, showing their accumulation in the IVD at different time points. (c) The measurement of H2 concentration in venous blood of IDD rats at 0 min, 20 min, 40 min, and 60 min after administration of AB@HPDA (*P* > 0.05, *n* = 5).

To verify the metabolism of AB@HPDA nanoparticles in the rat caudal disc, we injected Cy5.5-labeled AB@HPDA nanoparticles into the degenerated caudal disc. As shown in Figure 4, the fluorescence signal of AB@HPDA nanoparticles in the caudal disc decreased slowly until the nanoparticles were no longer present 13 days after injection (Figures 4(a) and 4(b)). In addition, we measured the concentration of H2 in the blood of IDD rats by gas chromatography. There was no significant difference in the content of H2 in the blood of rats between the AB@HPDA group and the normal group (*P* > 0.05, Figure 4(c)). The blood H2 level remained relatively low throughout the administration.

### 3.5. Treatment of AB@HPDA with IDD In Vivo

**Figure 5:**
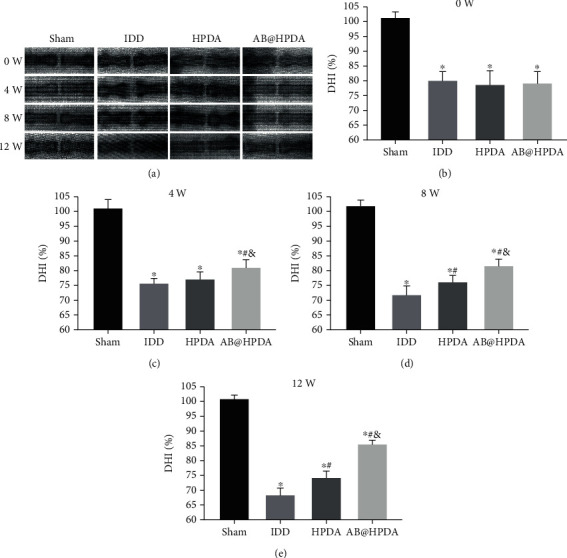
(a) Typical radiographs and (b–e) DHI ratio change of the effect of HPDA/AB@HPDA on IDD at 0, 4, 8, and 12 weeks after treatment. Data are presented here as the mean ± SD. ^∗^*P* < 0.05 vs. sham, ^#^*P* < 0.05 vs. IDD, ^&^*P* < 0.05 vs. HPDA (*n* = 10).

**Figure 6:**
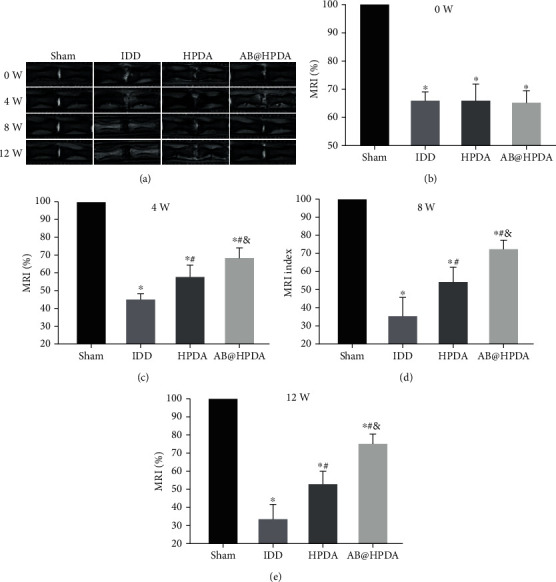
(a) Typical MRI and (b–e) signal intensity of MRI ratio change of the effect of HPDA/AB@HPDA on IDD at 0, 4, 8, and 12 weeks after treatment. Data are presented here as the mean ± SD. ^∗^*P* < 0.05 vs. sham, ^#^*P* < 0.05 vs. IDD, ^&^*P* < 0.05 vs. HPDA (*n* = 10).

**Figure 7:**
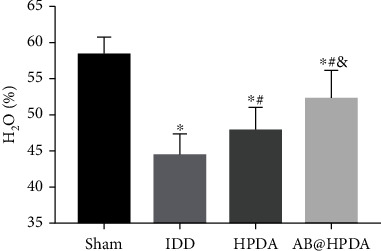
The water content change of HPDA/AB@HPDA with IDD at 12 weeks after treatment. Data are presented here as the mean ± SD. ^∗^*P* < 0.05 vs. sham, ^#^*P* < 0.05 vs. IDD, ^&^*P* < 0.05 vs. HPDA (*n* = 10).

**Figure 8:**
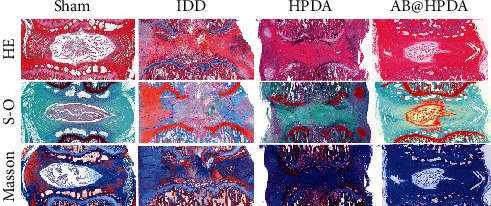
Typical HE, S-O and Masson staining of the tail discs at 12 weeks after HPDA/AB@HPDA treatment. Scale bar = 500 *μ*m.

**Figure 9:**
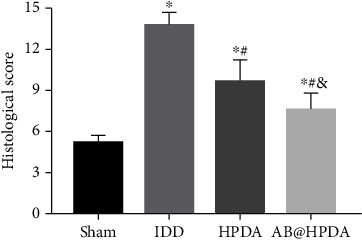
The histologic score at 12 weeks after HPDA/AB@HPDA treatment based on HE staining as shown in Figure 8. Data are presented here as the mean ± SD. ^∗^*P* < 0.05 vs. sham, ^#^*P* < 0.05 vs. IDD, ^&^*P* < 0.05 vs. HPDA (*n* = 5).

An animal model of IDD induced by acupuncture at the caudal vertebra was used. The effect of AB@HPDA in the treatment of IDD was tested in vivo. A 21G puncture needle was used for IVD puncture modeling. X-ray and MRI examinations showed that the DHI and signal intensity of the MRI ratio in the modeling group were significantly lower than those in the sham group after 2 weeks (*P* < 0.05, Figures 5 and 6). This proves that the acupuncture rat model of IDD was successfully established. After that, the groups were treated with saline, HPDA, and AB@HPDA. HPDA and AB@HPDA significantly decreased disc degeneration, as detected by X-ray (Figure 5), MRI (Figure 6), disc water content (Figure 7), and histology (Figures 8 and 9). Compared with the HPDA group, the effect of AB@HPDA was significantly enhanced, as detected by X-ray (Figure 5), MRI (Figure 6), disc water content (Figure 7), and histology (Figures 8 and 9).

The changes in DHI detected by X-ray in each group are shown in Figure 5. DHIs in the IDD group, HPDA group, and AB@HPDA group were significantly lower than those in the sham group at week 0 (*P* < 0.05). Compared with the IDD group, the DHI in the HPDA and AB@HPDA groups was significantly higher at 4, 8, and 12 weeks (*P* < 0.05). Compared with the HPDA group, DHIs in the AB@HPDA group were much higher at 4, 8, and 12 weeks (*P* < 0.05).

The changes in the structure and water content of the IVD detected by MRI are shown in Figure 6. As shown in Figure 6, the structure of the IVD in the sham group was intact, while in the other groups, the disc was damaged to varying degrees, accompanied by the T2 signal change of the disc. The signal intensity of the MRI ratio was detected by T2 signal change of the disc. Compared with the sham group, the signal intensities of the MRI ratios of the IDD group, HPDA group, and AB@HPDA group were significantly lower at 0, 4, 8, and 12 weeks (*P* < 0.05), respectively. The signal intensities of the MRI ratio in the HPDA group and AB@HPDA group were improved significantly compared with the IDD group (*P* < 0.05), while the signal intensities of the MRI ratios of the AB@HPDA group at 4, 8, and 12 weeks were much higher than those in the HPDA (*P* < 0.05). The water content changes of the IVD after 12 weeks are shown in Figure 7. HPDA and AB@HPDA significantly increased the water content of IVD compared with the IDD group. Compared with the HPDA group, AB@HPDA significantly increased the water content of the IVD (*P* < 0.05).

Histological changes in IVD were detected by HE, S-O, and Masson staining to study cell morphology and intervertebral disc matrix composition, respectively. As shown in Figure 9, in the sham group, the nucleus pulposus cells (NPCs) in the IVD were star-shaped and evenly dispersed in the NP tissue, and the boundary between the NP and the annulus fibrosus (AF) was clear. The purple area in H&E staining and the red area in S-O staining showed the proteoglycan matrix within the disc. The results showed that the proteoglycans were arranged in thin stripes. Compared with the sham group, the NP tissue in the IDD group was significantly reduced, and the NPCs became larger, rounder, and separated by dense areas of proteoglycan matrix, indicating severe IDD. HPDA and AB@HPDA can significantly reduce IDD by significantly improving the number of NPCs and the distribution of proteoglycans. Histological scores were used to assess the degree of IDD based on H&E staining results. The results showed that, compared with the Sham group, the IDD, HPDA, and AB@HPDA groups had significantly lower scores at 12 weeks (*P* < 0.05). The histological scores of the HPDA and AB@HPDA groups were significantly lower than those of the IDD group (*P* < 0.05), and the histological scores of the AB@HPDA group were significantly lower than those of the HPDA group (*P* < 0.05).

### 3.6. AB@HPDA Inhibits Disc ECM Degradation

**Figure 10:**
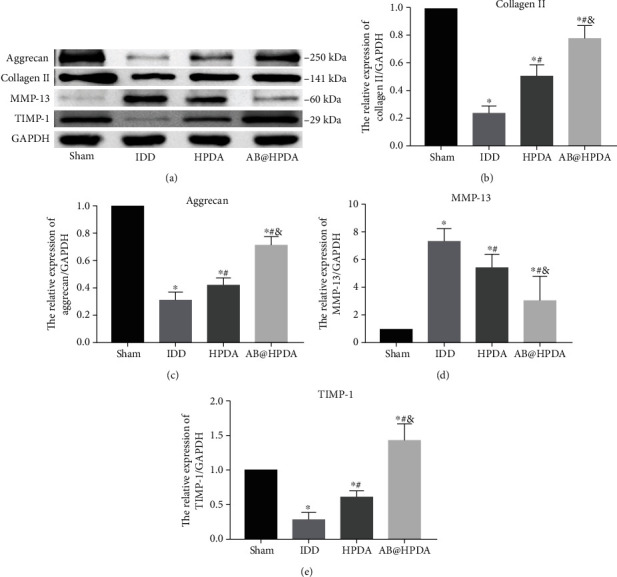
Effect of HPDA/AB@HPDA treatment on ECM-associated protein expression (*n* = 6). (a) WB analysis of collagen II, aggrecan, MMP-13, and TIMP-1 protein expression in NP tissue. Representative WB quantification of (b) collagen II, (c) aggrecan, (d) MMP-13, and (e) TIMP-1. Values are expressed as the mean ± SD, ^∗^*P* < 0.05 vs. sham, ^#^*P* < 0.05 vs. IDD, ^&^*P* < 0.05 vs. HPDA (*n* = 5).

The expression of major extracellular matrix (ECM) proteins (collagen II, aggrecan, MMP-13, and TIMP-1) in NP tissue was detected by WB at 12 weeks after HPDA/AB@HPDA treatment. As shown in Figure 10, compared with that in the sham group, collagen II, aggrecan, and TIMP-1 protein expression in the IDD group was significantly lower (*P* < 0.05), while MMP-13 expression was significantly higher (*P* < 0.05). HPDA and AB@HPDA significantly improved the expression of ECM-related proteins such as collagen II and aggrecan (*P* < 0.05). The contents of collagen II and aggrecan in the AB@HPDA group were significantly higher than those in the HPDA group (*P* < 0.05). By detecting the expression of ECM degradation-related proteins, it was found that AB@HPDA inhibited disc ECM degradation by significantly inhibiting the expression of MMP-13 protein and increasing the protein expression of TIMP-1 (*P* < 0.05).

### 3.7. AB@HPDA Alleviated Oxidative Stress and Inflammation after IDD

**Figure 11:**
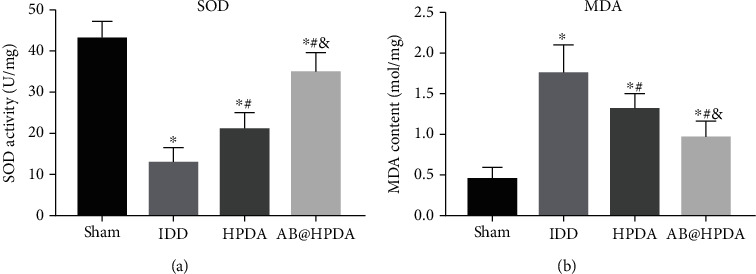
The level of oxidative stress in IDD was assessed by SOD and MDA. (a) SOD content of the NP tissue in the sham, IDD, HPDA, and AB@HPDA groups. (b) MDA level of NP tissue in the sham, IDD, HPDA, and AB@HPDA groups. ^∗^*P* < 0.05 vs. sham, ^#^*P* < 0.05 vs. IDD, ^&^*P* < 0.05 vs. HPDA (*n* = 5).

MDA and SOD levels were measured 12 weeks after IDD to determine the level of IDD oxidative stress (Figure 11). In the IDD group, the SOD level was increased significantly, and the MDA level was decreased significantly compared with that in the sham group. In the HPDA and AB@HPDA groups, the MDA level was decreased significantly and the SOD level was increased significantly compared with those in the IDD group (*P* < 0.05). In addition, the protective effect of AB@HPDA was better than that of HPDA (*P* < 0.05).

**Figure 12:**
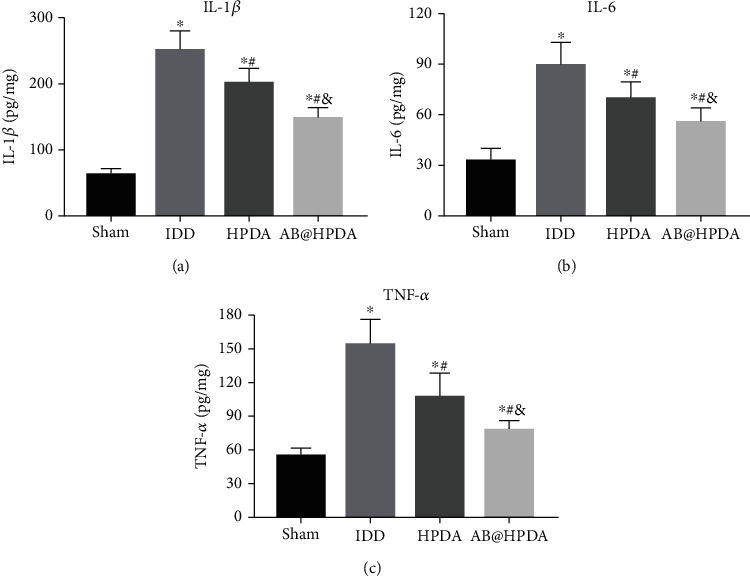
Determination of IL-1*β*, TNF-*α*, and IL-6 levels in the NP tissue by ELISA. The content of (a) TNF-*α*, (b) IL-1*β*, and (c) IL-6 in the sham, IDD, HPDA, and AB@HPDA groups. ^∗^*P* < 0.05 vs. sham, ^#^*P* < 0.05 vs. IDD, ^&^*P* < 0.05 vs. HPDA (*n* = 5).

The levels of IL-1*β*, TNF-*α*, and IL-6 were detected at 12 weeks after IDD to determine the IDD inflammatory levels by ELISA (Figure 12), and TNF-*α*, IL-1*β*, and IL-6 are major indicators of inflammation. The levels of TNF-*α*, IL-1*β*, and IL-6 in IDD rats were increased significantly compared with those in the sham group (*P* < 0.05). However, the TNF-*α*, IL-1*β*, and IL-6 contents were reduced significantly after treatment with HPDA and AB@HPDA (*P* < 0.05). In addition, TNF-*α*, IL-1*β*, and IL-6 levels were decreased significantly in the AB@HPDA group compared with those in the HPDA group (*P* < 0.05).

## 4. Discussion

A key problem hindering IDD treatment effects is maintaining a long-term effective drug concentration in the IVD with conventional drug delivery methods. To address this issue, we designed a pH-responsive AB@HPDA nanoparticle to deliver H2 according to different pH values in the IVD with different degrees of degeneration. To the best of our knowledge, this is the first report to treat IDD with pH-responsive H2-releasing nanomaterials according to the special acidic environment in the IVD. There are several advantages of this drug delivery system. First, AB@HPDA can achieve long-term high-concentration release of H2 in the IVD, which not only solves the disadvantage of low drug concentrations using the traditional drug delivery method but also prevents IDD caused by multiple punctures. Second, AB@HPDA reacts according to the special acidic environment of the IVD. In general, the more severe the IDD is, the lower the PH. AB@HPDA does not undergo significant decomposition in normal tissues (pH = 7.4), while it undergoes microdecomposition in normal IVD (pH = 7.1) and undergoes extensive decomposition in degenerated IVD (pH = 6.8, 6.5, 6.2). The H2 therapeutic effect is dose-dependent [33]. Theoretically, more H2 and better therapeutic effects could be obtained with the lower pH observed in degenerated IVDs. Our in vivo studies have shown that HPDA and AB@HPDA can effectively treat IDD by rebalancing oxidative stress and inflammation in IVD and restoring the stability of disc ECM. In vivo experiments also confirmed that HPDA has a good anti-inflammatory effect on IDD, and the anti-inflammatory effect of dopamine has been confirmed by many studies [34–36]. The combination of H2 and HPDA can significantly enhance the therapeutic effect of AB@HPDA. In conclusion, the present study has provided novel therapeutic targets for IDD by pH-responsive delivery of H2 through AB@HPDA. This AB@HPDA could control the release of H2 in response to the acidic pH conditions of degenerative IVDs.

IDD is a natural process of aging [37, 38]. Stress injury, smoking, hyperglycemia, iatrogenic injury, genetic differences, etc. can significantly increase the IDD process [39, 40]. The mechanics of the microenvironment can regulate cell fate, synthesis of the rigid extracellular matrix [41]. Conservative and surgical treatment to correct symptoms and structural abnormalities cannot completely repair degenerated discs. There are many studies on the treatment of intervertebral disc degeneration with good results [42–45]. The IVD is the largest avascular tissue in the human body and mainly relies on passive diffusion of the endplates for oxygen supply and nutrient metabolism. This leads to a special hypoxic [13], acidic [18], and hyperbaric [46] microenvironment in the IVD. The partial pressure of oxygen in the central area of the IVD is 1%[47]. As the IVD degenerates, the partial pressure of oxygen also decreases [48]. The pH in a healthy IVD is approximately 7.1[49]. The pH value can drop to 6.5 in severely degenerated IVD tissue, and it has even been reported that the pH value dropped to 5.5 in human degenerative disc surgery specimens [18, 27]. Recent studies have shown that pH has a certain effect on IDD [50]. NPCs are sensitive to pH changes in the ECM. At a certain low pH, the synthesis rate of the extracellular matrix decreases, and the degradation rate increases [51]. NPCs will die after prolonged exposure to this acidic environment [52]. The partial pressure of oxygen in the IVD is low, and the lactic acid produced by anaerobic metabolism will reduce the pH of the IVD [53]. Low pH can affect the synthesis of NPCs and the stability of ECM, which leads to IDD. Dysregulation of lactate transport and metabolism in the IVD can significantly increase the concentration of lactate, which in turn affects the acidity of the ECM. The effect of pH on NPCs is bidirectional. The ECM synthesis rate of NPCs is the fastest at pH 7.0-7.2, while the ECM synthesis rate decreases significantly in an acidic environment [17]. Low pH can significantly inhibit ECM-related protein synthesis [54]. The total amount of MMPs in IVD decreased significantly with decreasing pH. As the main factor degrading IVD collagen and inducing IDD, MMPs have a very obvious effect on NPCs. For TIMPs, the highest concentrations were found in the intervertebral disc and articular cartilage at pH 7.4. The acidic environment greatly reduces the content of TIMP-1, and its content at pH 6.4 is 3% of that at pH 7.4[17]. This finding suggests that the acidic environment in the IVD can significantly inhibit ECM synthesis in NPCs but cannot effectively prevent ECM catabolism. The acidic environment in degenerated IVD not only affects the synthesis and metabolism of matrix components but also significantly reduces the effect of cell transplantation in the treatment of IDD [27]. Transplanted cells, growth factors, and cytokines are very sensitive to changes in nutrient supply and metabolic microenvironment in IVD [55]. TGF-*β* can significantly increase the synthesis of glycosaminoglycans at pH 7.4, and this effect disappears when the pH value drops to 6.8, which can explain the poor repair effect of intradiscal injection of TGF-*β* [19]. The change in pH value in the IVD can not only affect nutritional metabolism but also further affect the expression and activity of NPCs as well as the effect of drug treatment. Changes in the IVD accelerate the process of degeneration, which further exacerbates the reduction in pH. To solve the problem of decreased drug activity due to the special acidic environment in the IVD, pH-responsive delivery of H2 through AB@HPDA nanoparticles was used for IDD treatment. H2 is a blunt gas with good anti-inflammatory, antioxidant, and antiapoptotic effects [56]. HPDA also has a good anti-inflammatory effect [34]. The activities of H2 and HPDA are not affected by special microenvironments such as acidity, hypoxia, and high pressure in the IVD. AB can release H2 quickly in acidic pH through an intramolecular cyclization reaction. However, due to its small molecular size, H2 is easily excreted from IVD, resulting in a low therapeutic effect. To overcome this disadvantage, AB@HPDA nanomaterials have acid-responsive release of H2, which is similar to AB nanomaterials. This enables AB@HPDA nanomaterials to realize H2 release in response to the acidic microenvironment in the degenerative IVD for efficient hydrogen therapy. In addition, the decomposition rate of AB in HPDA is greatly reduced compared with that of free AB. The H2 in AB@HPDA was rapidly released in the first 30 minutes, followed by a slow sustained release, and the H2 content peaked at approximately 24 hours and then decreased slowly. The early H2 rapid release effect is likely due to the AB loaded and absorbed on the nanoparticle surface. The sustained release of H2 may be attributed to the AB loaded in the nanoparticle core. As mentioned above, this segmented decomposition behavior benefits sustained hydrogen release (>1 day in vitro), which is very beneficial for long-term hydrogen therapy.

H2, a colorless, odorless small molecular gas with reductive properties, is considered a physiologically inert gas with no biological activity. Ohsava et al. reported that inhalation of 2% H2 can selectively scavenge hydroxyl radicals (OH) and peroxynitrite anions (ONOO-), which significantly improve cerebral ischemia–reperfusion injury in rats [22]. In recent years, an increasing number of studies have shown that H2 has selective antioxidant, anti-inflammatory, and antiapoptotic effects [57]. It has been widely used in disease models with good therapeutic effects, including diabetes, sepsis, atherosclerosis, hypertension, and cancer [58]. In recent years, the role of hydrogen in the treatment of bone and joint diseases has received increasing attention. It has been successfully applied to rheumatoid arthritis (RA) [59], osteoarthritis (OA) [60], osteoporosis [61], femoral head necrosis [62], and motor-related diseases [63]. H2 has many potential advantages for osteoarticular diseases. First, due to its extremely low molecular weight, electrical neutrality, and nonpolarity, H2 can easily penetrate cell membranes into mitochondria, the endoplasmic reticulum, and the nucleus. These organelles are the primary sites for the production of ROS, which can cause oxidative damage. Second, many studies have shown that H2 can exert therapeutic effects on osteoarticular diseases due to its selective antioxidant, anti-inflammatory, antiapoptotic effects, etc. Third, the current study revealed that H2 does not impair cells. H2 can exert its effects without interfering with normal cellular redox reactions and intracellular signaling. However, there are still some existing problems in H2 clinical use. Due to the low solubility and rapid release rate of hydrogen, it is difficult to maintain an effective therapeutic concentration, which restricts its application. This may be the reason why H2 therapy has not yet been used in IDD. Hence, we designed a pH-responsive H2 delivery system via AB@HPDA nanoparticles for IDD therapy. AB has a good H2 storage capacity (10 wt.%) and can generate H2 in an acid-triggered manner [32]. AB@HPDA nanoparticles were injected into the intervertebral disc at one time by local injection and metabolized in the intervertebral disc for up to 11 days (Figure 4). The constructed nanomedicine (AB@HPDA) exhibited an ultrahigh H2-loading capacity, high responsiveness to the acidic microenvironment in IVD for highly sustainable H2 release (>2 days), high therapeutic efficacy, and high biosafety.

There are several deficiencies in this experiment. First, we did not examine the timing and concentration of H2 release in the IVD. Limited by the current in vivo H2 detection technology, there is no effective method to detect the H2 content of the IVD. We attempted to indirectly monitor the H2 content in the IVD through the H2 content in the rat's blood. However, we did not find an obvious H2 peak. It is possible that the following two causes lead to this result. On the one hand, IVDs are closed spaces with slow metabolism and no blood circulation. Therefore, the H2 in the IVD cannot be released quickly into the blood, especially in the tail. On the other hand, AB@HPDA nanoparticles were proven to be pH-responsive. AB@HPDA nanoparticles have a slow and controlled release effect without the rapid release of large amounts of H2. As a result, the H2 in the blood is at a lower level. We speculated that due to the special microenvironment in the intervertebral disc, AB@HPDA nanoparticles under the same pH conditions should be released slower and longer than those in the in vitro environment. However, extensive experimental and clinical validation is still needed. In this experiment, we only studied the metabolism of AB@HPDA, which is considered to reflect its H2 release in vivo. Second, recent studies have shown the therapeutic effect of hydrogen on IDD, but the mechanism of action is unclear. The specific molecular mechanisms involved in this process require further basic research. Third, rats were used in this study because the operation is simple, safe, and reproducible using rat IDD modeling technology. However, there are differences between humans and rats in the acidic environment of the IVD. Further studies are needed to confirm the therapeutic effect of H2 in humans.

## 5. Conclusion

In conclusion, we reported a novel strategy to treat IDD by encapsulating a pH-responsive delivery of AB into HPDA. We designed and manufactured pH-responsive H2 delivery through AB@HPDA for IDD therapy according to the different pH values in the IVD with different degrees of degeneration. AB@HPDA can achieve long-term controlled release of H2 in IVD based on different degrees of degeneration and pH values, which can effectively treat IDD. The mechanism of AB@HPDA may be to rebalance oxidative stress and inflammation in degenerative IVDs. AB@HPDA can restore the stability of disc ECM by upregulating the expression of anabolic proteins (such as collagen II, aggrecan, and TIMP-1) and downregulating the breakdown expression of metabolic proteins such as MMP-13 for the recovery of IDD. Delivery of H2 by AB@HPDA nanoparticles is an effective strategy for IDD treatment. Therefore, this study provides a potential therapeutic option for IDD therapy and may have promising applications in clinical research in the near future.

## Data Availability

The data used to support the findings of this study are included within the article.

## Supplementary Materials

Supplementary Materials Figure S1: HAADF-STEM-EDS images of AB@HPDA nanoparticles. The element maps showed the distribution of C (red), N (blue), O (green), and B (yellow).
