# Supplementary material for: pH-Responsive Delivery of H2 through Ammonia Borane-Loaded Hollow Polydopamine for Intervertebral Disc Degeneration Therapy
Source: Oxid Med Cell Longev. 2023 Feb 2;2023:7773609. doi: 10.1155/2023/7773609 (PMC9911255; doi:10.1155/2023/7773609)
Supplement: Supplementary Materials — Figure S1: HAADF-STEM-EDS images of AB@HPDA nanoparticles. The element maps showed the distribution of C (red), N (blue), O (green), and B (yellow). [file 7773609.f1.docx]

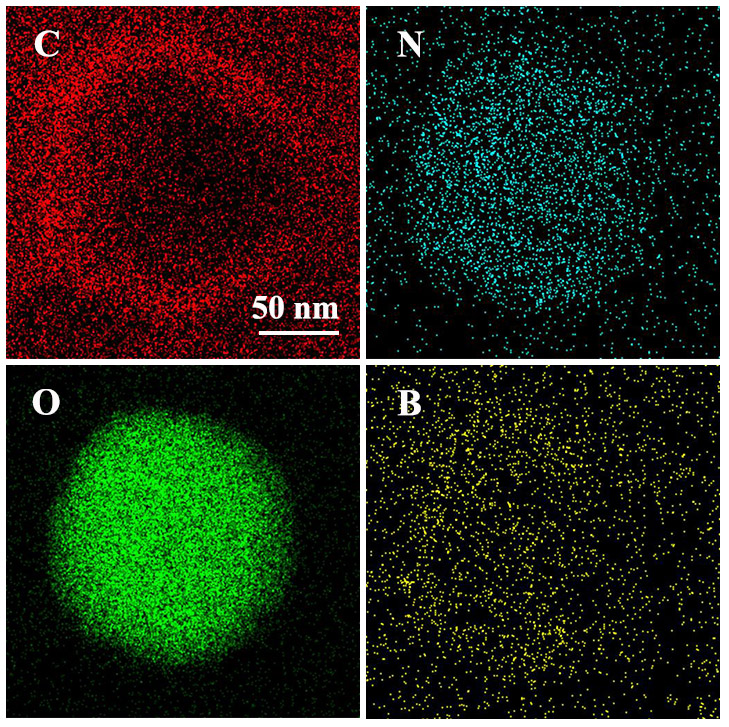


FIGURE S1: HAADF-STEM-EDS images of AB@HPDA nanoparticles. The element maps showed the distribution of C (red), N (blue), O (green) and B (yellow).
